# Development and Validation of Engineering Professional Moral Identity Measure

**DOI:** 10.1007/s11948-026-00593-0

**Published:** 2026-04-11

**Authors:** Dayoung Kim, Bailey McOwen

**Affiliations:** Department of Engineering Education, Virginia Tech, Data & Decision Sciences (D&DS) Building 336, 727 Prices Fork Rd, Blacksburg, VA 24061 USA

**Keywords:** Engineering ethics, Moral identity, Measurement, Assessment

## Abstract

Engineering ethics education often emphasizes teaching reasoning skills while overlooking other influential dimensions on one’s ethical behavior – e.g., whether students see ethical responsibility as part of who they are as professionals. One of the challenges in integrating such overlooked dimensions in engineering ethics education is the limited resources in assessing student outcomes. To address this challenge, adapting a widely used moral identity scale to the engineering context, this paper introduces an initial development and validation effort of the Engineering Professional Moral Identity (EPMI) instrument, designed to assess the extent to which ethical responsibility is integrated into one’s identity as an engineer. Exploratory and confirmatory analyses were performed with survey data from 515 practicing engineers. Results supported a two-factor structure, internalization and symbolization, with an eight-item model. We argue that EPMI complements reasoning-focused measures by capturing the identity dimension of ethical development, which enables longitudinal tracking of students’ identity development and targeted instruction. Findings from this study provide initial validity evidence for EPMI and position the tool as a practical tool for assessing and cultivating identity-centered ethics education in engineering education.

## Introduction

In recent decades, engineering educators and professional organizations have called for stronger integration of ethics into engineering curricula, emphasizing students’ ability to understand ethical issues associated with engineering work and make ethical judgment (CEE 2016; ABET, 2025). Despite widespread agreement on the importance of ethics education, assessments often focus on students’ knowledge of ethical codes or their reasoning and judgment based on responses to case studies, failing to capture other important dimensions of ethical behavior, such as moral identity (Kim & Bairaktarova, 2023). The main reason behind the failure could be what’s currently required in the engineering curriculum – ABET criterion on ethics emphasizes students’ ability to make ethical judgment. However, ethics in engineering is not just a cognitive task as the practice is embedded in personal values, professional roles, and sociotechnical systems (Whitbeck, 2011). Such a nature of engineering ethics has led scholars to argue that cultivating ethical engineers requires going beyond focusing on cognitive abilities, such as knowledge and reasoning. Cultivating ethical engineers needs to consider the moral self-concept students build into their professional formation (Hess et al., 2024; Hess & Fore, 2018; Kim, 2022a).

Yet, despite this recognition, there remains no validated instrument for assessing engineering-specific moral identity in professional contexts. This gap is significant because while cultivating moral identity is viewed as central to the professional formation of engineers, current assessments do not capture this dimension. Instructional design benefits from aligned assessment; therefore, a measure that captures engineers’ moral identity would enable the development and evaluation of identity-centered ethics curricula (Streveler et al., 2012).

The Engineering Professional Moral Identity (EPMI) instrument offers a promising approach for addressing this gap. Developed to measure the extent to which students internalize ethics as a core part of their identity as engineers, EPMI shifts the focus from what students know to who they are becoming as professionals. EPMI aligns with evidence suggesting that individuals are more likely to act ethically when moral values are central to their self-concept (Aquino & Reed, 2002; Damon & Hart, 1992). As Hardy and Carlo have pointed out, improvements and expansions of the methodologies used in moral identity research are critically needed (Hardy & Carlo, 2011). In particular, tools that can be adapted to specific disciplinary contexts like engineering, which present unique moral and epistemic challenges, should be developed. Developing an instrument to assess moral identity from an engineering-specific perspective provides the opportunity to capture a fuller understanding of how engineers internalize and enact moral values within their profession and can ultimately guide necessary ethics curricular design and innovation.

Building on the argument that moral identity is a key driver of moral behavior, this paper reports on the development, validation, and implications of the EPMI instrument as a field-specific measure of moral identity for the engineering profession. The sections that follow first review the theoretical foundations of moral identity and its relevance to engineering education. We then describe the construction of the EPMI instrument, present evidence from exploratory and confirmatory factor analyses, and discuss implications for assessment and pedagogy. Through this work, we aim to demonstrate how EPMI can provide educators with deeper insight into students’ ethical development, complement traditional assessment tools, and ultimately support more intentional, identity-centered approaches to ethics instruction. Throughout this paper, we answer the following research question: *RQ) What evidence supports the validity and reliability of the newly developed Engineering Professional Moral Identity (EPMI) measure?*

## Literature Review

### Moral Identity

#### Concept

While it has been assumed that mature moral reasoning leads to one’s moral action (e.g., Kohlberg, 1969), arguments have been made that suggest reasoning does not necessarily lead to one’s moral behavior (Blasi, 1980; Haidt, 2001). The concept of *moral identity* has emerged from the investigation for bridging the gap between moral reasoning and behavior. Among moral psychologists, moral identity has been defined as “the degree to which being a moral person is important to a person’s identity” (Hardy & Carlo, 2011, p. 212).

Hardy and Carlo (2005) agree that cognitive and emotional factors cannot fully explain moral action and introduce moral identity as an important construct for predicting moral behavior: “when morality is important and central to one’s sense of self and identity, it heightens one’s sense of obligation and responsibility to live consistent with one’s moral concerns” (p. 234). Such explanations align with identity-based motivation theory by Oyserman and Destin (2010), which argues that people “prefer identity-congruent to identity-incongruent actions” (p. 1001). Also, when one feels a behavior is identity-congruent, they interpret difficulties in engaging in the behavior as “important not impossible, and therefore effort is meaningful not pointless” (p. 1002). Therefore, one’s perception of identity-congruence becomes an important source of persistent behavior, even in the moral domain.

The initial idea of moral identity was suggested by Blasi (1999), who argued that the self is a critically important factor in one’s moral behavior. His Self-Model of moral action (Blasi, 1983) suggests two important factors: a sense of personal responsibility and a drive for self-consistency. Once a person makes a moral judgment, they conduct a responsibility judgment through which they evaluate if the morally right action derived from their own judgment is strictly necessary for the self. The more the moral consideration is regarded as essential to the self, the more people tend to act in accordance with their judgment (Narvaez & Lapsley, 2009). Blasi explains this tendency toward self-consistency as a bridge between cognition and action. According to Blasi, different people may have different moral aspects that characterize the self, and moral identity captures these individual differences (Blasi, 1983).

#### Moral Identity and Moral Behavior

Since the concept of moral identity stemmed from the need to fill the missing link between moral judgment and behavior, how moral identity relates to moral behavior has been an important topic of study. While moral behavior was operationalized and measured differently across different studies, general findings showed that moral identity is a significant predictor of one’s moral behavior. For example, Aquino and Reed (2002) showed that one’s moral identity is a significant predictor of one’s donation behavior, which was recorded by an external observer. Hardy (2006) reported that one’s prosocial identity is positively associated with one’s prosocial behavior, measured by the 25-item self-report measure of prosocial behavior tendencies. Hertz and Krettenauer (2016)’s meta-analysis of 111 studies examining the relationships between moral identity and moral behavior showed that moral identity clearly predicts moral behavior, although it does not appear to be an extraordinarily strong predictor according to the effect sizes.

#### Existing Measures of Moral Identity

Researchers have developed measurement instruments for moral identity. Among those, Aquino and Reed (2002)’s measure, called Moral Identity Scale, has been the most widely used measure. Aquino and Reed’s measure presents nine words of moral traits (caring, compassionate, fair, friendly, generous, helpful, hardworking, honest, and kind) and asks survey-takers to respond to ten items, each of which falls under one of two factors: internalization and symbolization. Among the ten items, five items are for measuring moral identity internalization, which represents the extent to which having the moral traits are important part of who they are as a person. The other five items are for measuring moral identity symbolization, which represents the extent to which they present themselves as individuals with those traits. Strengths of this measure include its strong theoretical grounding in moral identity theory and its wide use across diverse populations, setting a strong precedent. However, a notable limitation is that the scale’s moral trait list and item language are context-neutral, which make it less suitable for professional or discipline-specific settings such as engineering.

Barriga et al. (2001)’s Adapted Good-Self Assessment (GSA) has also been used to measure prosocial identity (Hardy, 2006). Survey-takers are asked to rate 16 virtues, including moral virtues (considerate, honest, helpful, sympathetic, generous, sincere, fair, and dependable) and non-moral virtues (imaginative, industrious, outgoing, athletic, funny, logical, independent, and energetic) on a scale of 1 (not important to me) to 4 (very important to me). Then the prosocial identity is calculated by subtracting the mean score of the non-moral virtues from the mean score of the moral virtues. The GSA offers a straightforward comparison between moral and non-moral virtues, which provides a useful differentiation of prosocial identity. However, its simplicity comes at the cost of depth as it is more concerned with moral valuation than actually assessing the internalization or expression of moral identity.

Black and Reynolds (2016) developed a 20-item measure of moral identity, called Moral Identity Questionnaire (MIQ), which consists of two factors of moral self (the importance one gives to their moral principles) and moral integrity (the importance one gives to acting according to their moral principles). The moral self is measured by 8 among the 20 items, and the moral integrity is measured by 12 among the 20 items. The answer choices are on a 6-point Likert scale (strongly disagree to strongly agree). For example, survey-takers are asked to rate statements like “It is important for me to treat other people fairly,” and “Not hurting other people is one of the rules I live by,” from 1 to 6. The MIQ provides rich coverage of integrity and moral self-importance, but it is not contextually grounded, which limits its transferability to specialized disciplines such as engineering, where ethical obligations are embedded in the professional roles and standards.

Goranson et al. (2022) developed a measure called Moral Identity Picture Scale (MIPS), which uses a series of pictures to measure a person’s moral identity. This was achieved by arguing that existing moral identity scales only focus on measuring only partial elements of morality, such as self-perceived goodness, and cannot capture a fuller scope of moral identity. Based on the Theory of Dyadic Morality, which argues that moral roles contain not only doers/agents of moral/immoral acts (heroes and villains) but also recipients/patients (victims and beneficiaries), they created 16 pictures of different moral scenarios. Among those four depict a hero and villain, four depict a hero and beneficiary, four depict a villain and victim, and the remaining four depict a victim and beneficiary. After seeing each picture, survey-takers are asked to answer the question “How much do you identify with each person above?” on a 1 (not at all) to 4 (extremely) Likert Scale. The MIPS is innovative in its visual approach and its expansion beyond trait-based measures to include empathy with moral agents and patients. However, the format is less adaptable for professional contexts that require domain-specific language and scenarios. Moreover, its qualitative interpretive demands make large-scale quantitative validation in specialized populations, such as engineering, more difficult.

Overall, there has been little study that focused on moral identity in specific contexts like engineering, and there is no validated instrument to measure moral identity in engineering-specific contexts. Since ethics in professional contexts goes beyond general morality and requires understanding of specific professional values, norms, and principles, a measure that is tailored to the engineering context and validated with engineering professionals is needed to properly measure engineers’ moral identity in professional contexts. Ultimately, Aquino and Reed’s (2002) measure was chosen to adapt because it provides a well-validated, theoretically grounded framework that directly operationalizes moral identity through its dual focus on internalization and symbolization. This strong foundation and conceptual clarity made it an ideal starting point for adaptation to the engineering context.

### Engineering Professional Moral Identity

#### Concept

Blasi (1983, 1999) and Hardy and Carlo (2011) identify moral identity as a critical mechanism that links moral reasoning with moral behavior. In the context of engineering, where decisions frequently carry significant implications for public safety, environmental sustainability, and societal welfare, moral identity may likewise serve to bridge the gap between ethical judgment and ethical behavior. However, because engineering presents unique professional contexts and accompanying unique ethical considerations, it would be helpful to define a specific concept that can link engineers’ ethical judgment and behavior: *engineering professional moral identity*. Reflecting how general moral identity has been defined in Hardy and Carlo (2011), engineering professional moral identity can be defined as the degree to which being an ethical engineer is important to their professional identity as an engineer.

Although no studies to date have explicitly defined or examined this concept, existing literature on professional identity and ethics in engineering and engineering education provides a foundation for its development. For example, Downey et al. (2007) argue that issues in engineering ethics cannot be separated from the identity of engineers and their accompanying responsibilities. Their study shows that ethics programs evolve as societies redefine what it means to be an engineer and what responsibilities engineers should carry. For example, in Germany, ethics became tied to rebuilding an identity of engineers as socially responsible after World War II. In France, the tradition of prestigious higher-education institutions has downplayed ethics as engineers have been assumed to be inherently moral. This work ultimately demonstrates how ethics education emerges as part of the broader formation of professional identity, providing the initial link between engineering ethics and engineering identity.

Martin et al. (2021) also highlight that ethics in engineering cannot be fully addressed through individual moral reasoning alone; engineers’ ethical actions are often influenced by their roles, organizational cultures, and broader institutional systems. Because ethical challenges frequently arise from these external factors, ethics in engineering must be understood as socially and professionally situated. Rodriguez et al. (2018) highlighted how engineering identity is formed through a dynamic and socially constructed process; this suggests that engineers’ moral identity can be strengthened when ethical values are embedded in the norms and expectations of the engineering community. What counts as important ethical considerations of engineers may align with engineers’ perceptions of who engineers are and what their roles should be. This may be because individuals are more likely to persist in moral action, even in the face of professional and organizational barriers, when they perceive such ethical behavior as consistent with their identity (Oyserman & Destin, 2010; Blasi, 1983, 1999).

Taken together, these perspectives reinforce the idea that professional ethical behavior of engineers has an element of internalization of values that are upheld within the professional community. This idea informs the conceptual basis for engineering professional moral identity and underscores its potential role in strengthening ethical practice within the profession.

#### Importance in Engineering Ethics Education

Traditional engineering ethics curricula have emphasized principle-based reasoning and case study analysis, and this approach may have limitations in effectively bridging the gap between students’ knowledge of engineering ethics and their moral actions (Bairaktarova & Woodcock, 2015; Herkert, 2000; Lawlor, 2021; Kim & Jesiek, 2019). Integrating moral identity into engineering ethics education is essential for forming engineers who both understand ethical principles and view it as central to their professional identity (Stappenbelt, 2013). This is because individuals differ in the degree to which moral traits are central to their self-concept, a characteristic called moral identity internalization (Aquino & Reed, 2002).

People with higher levels of moral identity (those who show a higher level of internalization of moral values) are more likely to engage in moral behavior across various contexts, even when external incentives are absent (Aquino & Reed, 2002). By fostering engineering-specific moral identities in students, we anticipate that their sense of moral obligation and personal responsibility can be enhanced (Loui, 2005). This can eventually lead to effectively bridging students’ knowledge of engineering ethics and their moral actions, which is essential for working as an engineer who can consider normative purposes of engineering work and take actions (Katz, 2011; Kim & Jesiek, 2023).

## Methods

### Measurement Development

Among the measures of moral identity, we decided to utilize Aquino and Reed (2002)’s moral identity measure as a basis to develop an engineering professional moral identity measure. It was because Aquino and Reed’s measure is the most well-known and widely used measure of moral identity. As discussed in the literature review section, Aquino and Reed’s measure consists of 10 items with two factors, each of which represents the degree to which one internalizes and symbolizes the characteristics of a moral person. The instruction of the measure lists nine words that characterize a person who can be considered as a moral person and asks the survey takers to answer the following 10 items based on their reflection on the words. Table 1 shows the original moral identity measure of Aquino and Reed (2002).

**Table 1:** Original moral identity measure (Aquino & Reed, 2002)

Instruction: Listed below are some characteristics that may describe a person: caring, compassionate, fair, friendly, generous, helpful, hardworking, honest, and kind The person with these characteristics could be you or it could be someone else. For a moment, visualize in your mind the kind of person who has these characteristics. Imagine how that person would think, feel, and act. When you have a clear image of what this person would be like, answer the following questions.	
**Q#**	**Statement**
**1**	It would make me feel good to be a person who has these characteristics.
**2**	Being someone who has these characteristics is an important part of who I am.
**3**	I often wear clothes that identify me as having these characteristics.
**4**	I would be ashamed to be a person who has these characteristics.
**5**	The types of things I do in my spare time (e.g., hobbies) clearly identify me as having these characteristics.
**6**	The kinds of books and magazines that I read identify me as having these characteristics.
**7**	Having these characteristics is not really important for me.
**8**	The fact that I have these characteristics is communicated to others by my membership in certain organizations.
**9**	I am actively involved in activities that communicate to others that I have these characteristics.
**10**	I strongly desire to have these characteristics.

To adopt the measure to better fit with engineering contexts and be able to actually measure engineering professional moral identity, we decided to modify the words in the instrument. We first changed all the “person” in the measure to specifically “engineer.” To modify the words that characterize a moral person into engineering-specific words, we reviewed several codes of ethics of engineers, including NSPE, IEEE, AIChE, and ASCE, as well as the engineering virtues list suggested by Pritchard (1998), and identified ten different words that were well represented across the codes and the list. Those included *honest; high integrity; cooperative; loyal; conscientious; objective; technically competent; fair-minded; committed to quality; open to criticism; committed to public health*,* safety*,* and welfare*.

While we mostly followed the exact language of the original moral identity measure to draft each item of the measure, we also adjusted some language in the items to better fit with engineering contexts. For example, as an example in the parenthesis in item 5, we added “volunteering” too, as it is a common activity that engineers often participate in with their expertise. Some items were also modified just for clarity or recency. For example, the third item “I often wear clothes…” was changed to “I often dress in ways that…” and the sixth item “The kinds of books and magazines that I read…” was changed to “The kinds of media that I consume…”. Table 2 shows the modified version of the moral identity measure, namely, the Engineering Professional Moral Identity (EPMI) measure.

**Table 2:** 10-Item EPMI

Instruction: We are interested in how engineers and other technical professionals think about who they are. Listed below are some characteristics that might describe an engineer: honest; high integrity; cooperative; loyal; conscientious; objective; technically competent; fair-minded; committed to quality; open to criticism; committed to public health, safety, and welfare The engineer with these characteristics could be you or it could be someone else. For a moment, visualize in your mind the kind of engineer who has these characteristics. Imagine how that engineer would think, feel, and act. When you have a clear image of what this engineer would be like, answer the following questions.		
**Q#**	**Statement**	**Factor**
**Q1**	It would make me feel good to be an engineer who has these characteristics.	Internalization
**Q2**	Being an engineer who has these characteristics is an important part of who I am.	Internalization
**Q3**	I often dress in ways that identify me as having these characteristics.	Symbolization
**Q4**	I would be ashamed to be an engineer who has these characteristics.	Internalization
**Q5**	The types of things I do in my spare time (e.g., hobbies, volunteering) clearly identify me as having these characteristics.	Symbolization
**Q6**	The kinds of media I consume identify me as having these characteristics.	Symbolization
**Q7**	Having these characteristics is not really important to me.	Internalization
**Q8**	The fact that I have these characteristics is communicated to others by my membership in certain organizations.	Symbolization
**Q9**	I am actively involved in activities that communicate to others that I have these characteristics.	Symbolization
**Q10**	I strongly desire to have these characteristics.	Internalization

## Data Collection

The survey was distributed to engineering practitioners through various channels, including social media (e.g., LinkedIn, Twitter) and alumni associations of the lead author’s and the lead author’s colleagues’ institutions in the fall of 2020. Data was collected via Qualtrics. As the data of this study was collected as part of the larger study (Kim, 2022b), the survey also included other instruments and was designed to be completed in approximately 20 min. We had four recruitment criteria: (1) holding at least one academic degree in engineering, technology, or a related field, (2) received BS degree more than 3 years ago (graduated before September 2017), (3) currently working full time in industry as an engineer or other technical professional (including management) or currently unemployed but recently worked full time in industry as an engineer or other technical professional, and (4) current country of residence is the United States. At the end of the survey, the participants could voluntarily leave their name and email address to be entered into a drawing for a $100 gift card (with 1 in 20 odds of winning). As a result, 651 practicing engineers at least partially completed the survey, and after the data cleaning, 515 survey responses could be used in this study.

### Data Analysis

To examine the validity evidence of the measure, we conducted an exploratory factor analysis (EFA) followed by a confirmatory factor analysis (CFA). To conduct both analyses with a single administration of the survey, we randomly split the sample in half and conducted the EFA with the first half of the sample and the CFA with the second half of the sample (Lee et al., 2018; Lorenzo-Seva, 2022).

#### Exploratory Factor Analysis

Before conducting the EFA with the first half of the sample, we first checked the Kaiser–Meyer–Olkin (KMO) measure of sampling adequacy (Kaiser, 1970). Then we checked whether our data meets the multivariate normality assumption of the maximum likelihood (ML) factor analysis. For that, we first examined whether the absolute value of the skewness of each item was below 2.0 and that of the kurtosis of each item was below 7.0 by using the “moments” library in R, and then conducted Mardia’s test by using the “MVN” library in R.

After checking the multivariate normality assumption, we examined the internal consistency of the items. By using the “Hmisc” library in R, we checked whether the items that were designed to measure the same factors of the EPMI were significantly correlated with each other. Then we calculated Cronbach’s alpha value to evaluate the overall internal consistency of the measure based on the correlation matrix.

After checking the normality assumption and the correlations, we performed the EFA. For the EFA, we used the promax (oblique or non-orthogonal) rotation, as it is considered to provide a more realistic representation of the phenomena of interest in the social science research (Fabrigar et al., 1999). We determined the number of factors by using both the scree plot and parallel analysis.

#### Confirmatory Factor Analysis

Similar to the EFA, before conducting the CFA, we first checked the Kaiser–Meyer–Olkin (KMO) measure of sampling adequacy, and the multivariate normality assumption of the maximum likelihood (ML) factor analysis. Then we also examined whether the items that were designed to measure the same factors of the EPMI were significantly correlated with each other and the internal consistency with Cronbach’s alpha value.

After checking the normality assumption and the correlations, we performed the CFA. We first started from the model that we obtained based on the EFA and tested if the model shows satisfactory fit statistics. To evaluate the model fit, we used a combination of multiple fit indices, including the Comparative Fit Index (CFI), Tucker Lewis Index (TLI), and Root Mean Square Error of Approximation (RMSEA). To determine if an addition or deletion of certain paths on the model could improve the model fit, we also checked the modification indices.

## Results

### Results of the Exploratory Factor Analysis

The KMO was 0.71, which indicates a middling level of data for conducting the factor analysis (Kaiser, 1970, 1974). The skewness and the kurtosis of the items written for the Internalization factor (Q1, Q2, Q4, Q7, and Q10) were beyond the range for arguing univariate normality (below the upper limit of 2 for skewness and the upper limit of 7 for kurtosis). Especially, Q1 had a skewness of -4.26 and kurtosis of 23.54, and Q4 had a skewness of -4.29 and kurtosis of 18.62. This indicates a severe violation of the normality assumption for those two items.

For Q2, Q7, and Q10, although their skewness and kurtosis exceeded the threshold values, the values were still close to the limit. The skewness and kurtosis of the items written for the symbolization factor (Q3, Q5, Q6, Q8, and Q9) ranged between − 0.71 and − 0.17 for the skewness and between − 0.91 and − 0.15 for the kurtosis, which suggests that the items satisfy the univariate normality. Table 3 shows the skewness and kurtosis of items for each factor. The Mardia’s test was significant, which means the multivariate normality assumption was violated. Therefore, we decided to conduct the EFA with the ordinary least squares estimator (Revelle, 2014).

**Table 3:** Skewness and Kurtosis of Items for Each Factor

Item	Skewness	Kurtosis	Item	Skewness	Kurtosis
**Q1**	-4.26	23.54	**Q3**	-0.17	-0.75
**Q2**	-2.51	8.88	**Q5**	-0.71	-0.15
**Q4**	-4.29	18.62	**Q6**	-0.47	-0.28
**Q7**	-2.36	5.29	**Q8**	-0.31	-0.91
**Q10**	-2.08	5.22	**Q9**	-0.55	-0.43

Then we calculated the correlation matrix and Cronbach’s alpha. Table 4 shows the correlation matrix. Most items in the same factor were significantly correlated with each other, but Q4 was not significantly correlated to Q7. Therefore, based on the examination of the normality assumption and the correlation matrix, we decided to drop Q1 and Q4, because (1) participants’ answers to both Q1 and Q4 severely deviated from the normal distribution, and (2) Q4 did not correlate with one of the other items in the same factor (Internalization). After the removal, the overall Cronbach’s alpha was 0.73, which is considered an acceptable level of internal consistency (Nunnally & Bernstein, 1994). After the correlation tests, we moved on to conduct the EFA.

**Table 4:** Correlation matrices for each factor

Internalization					Symbolization				
	Q1	Q2	Q4	Q7		Q3	Q5	Q6	Q8
Q1					Q3				
**Q2**	0.62^***^				**Q5**	0.33^***^			
**Q4**	0.32^***^	0.18^**^			**Q6**	0.35^***^	0.40^***^		
**Q7**	0.15^*^	0.24^***^	0.08		**Q8**	0.29^***^	0.35^***^	0.37^***^	
**Q10**	0.25^***^	0.30^***^	0.26^***^	0.31^***^	**Q9**	0.20^**^	0.46^***^	0.36^***^	0.52^***^

For the number of factors for the EFA, both scree plot and the parallel analysis suggested two factors, well aligned with the theorized number of factors. Then we ran the EFA with the 8-item EPMI. Overall, the results of the EFA suggested an 8-item survey with a 2-factor structure as reported in Table 5. Although factor loadings for some items are somewhat low (especially, Q2), all items had loadings of at least 0.32 on the theorized factor, and there were no cross-loading items (each item’s loading on the other factor is less than half of the main factor loading). Therefore, we decided to proceed with the analysis while being careful about interpretation of our results.

**Table 5:** 2-factor structure of 8-item EPMI (only factor loadings larger than 0.10 are presented in the table; Factor 1 = Internalization, Factor 2 = Symbolization)

Item	Statement	Factor 1	Factor 2
**Q2**	Being an engineer who has these characteristics is an important part of who I am.	**0.38**	0.18
**Q7**	Having these characteristics is not really important to me.	**0.76**	-0.26
**Q10**	I strongly desire to have these characteristics.	**0.45**	0.22
**Q3**	I often dress in ways that identify me as having these characteristics.	0.11	**0.40**
**Q5**	The types of things I do in my spare time (e.g., hobbies, volunteering) clearly identify me as having these characteristics.		**0.62**
**Q6**	The kinds of media I consume identify me as having these characteristics.		**0.62**
**Q8**	The fact that I have these characteristics is communicated to others by my membership in certain organizations.		**0.77**
**Q9**	I am actively involved in activities that communicate to others that I have these characteristics.		**0.67**

### Results of the Confirmatory Factor Analysis

The KMO was 0.72, which indicates a middling level of data for conducting the factor analysis (Kaiser, 1974). The skewness and the kurtosis of the items written for the Internalization factor (Q2, Q7, and Q10) ranged between − 2.23 and − 1.46 for the skewness and between 1.84 and 7.83 for the kurtosis, which suggest the values generally satisfy the univariate normality, while careful attention needs to be paid to Q2, which had the skewness of -2.23 and the kurtosis of 7.83.

The skewness and kurtosis of the items written for the symbolization factor (Q3, Q5, Q6, Q8, and Q9) ranged between − 0.61 and − 0.22 for the skewness and between − 0.82 and − 0.12 for the kurtosis, which suggests that the items satisfy the univariate normality. Table 6 shows the skewness and kurtosis of items for each factor. The Mardia’s test was significant, which means the multivariate normality assumption was violated. Therefore, we decided to conduct the robust maximum likelihood CFA with the Satorra-Bentler corrections.

**Table 6:** Skewness and Kurtosis of Items for Each Factor

Item	Skewness	Kurtosis	Item	Skewness	Kurtosis
**Q2**	-2.23	7.83	**Q3**	-0.22	-0.82
**Q7**	-2.00	4.55	**Q5**	-0.61	-0.12
**Q10**	-1.46	1.84	**Q6**	-0.39	-0.27
			**Q8**	-0.37	-0.82
			**Q9**	-0.48	-0.64

We calculated the correlation matrix and Cronbach’s alpha. Table 7 shows the correlation matrix. The overall Cronbach’s alpha was 0.73, which is considered an acceptable level of internal consistency (Nunnally & Bernstein, 1994).

**Table 7:** Correlation Matrices for Each Factor

Internalization			Symbolization				
	Q2	Q7		Q3	Q5	Q6	Q8
Q2			Q3				
**Q7**	0.31^***^		**Q5**	0.30^***^			
**Q10**	0.45^***^	0.43^**^	**Q6**	0.22^***^	0.53^***^		
			**Q8**	0.34^***^	0.33^***^	0.28^***^	
			**Q9**	0.29^***^	0.44^***^	0.33^***^	0.63^***^

Finally, we ran the confirmatory factor analysis with the 8-item 2-factor structure model identified based on the EFA results (Model 1 in Table 8). We checked whether the data fit the model with multiple fit indices, including CFI, TLI, and RMSEA. Table 8 shows the fit indices for Model 1. According to the results, the data did not fit the model satisfactorily. Therefore, we checked the modification indices to explore how we could improve the model. As a result, we found that the modification index for making Q8 and Q9 covary was 49.91, which is significantly higher than the cutoff value of 3.82. Since those two items belong to the same factor (symbolization), we decided to allow them to covary. After the modification, we tested the modified model (Model 2) and found that the fit indices for Model 2 were improved: Model 2 showed acceptable fit with the data.

**Table 8:** Fit parameters for the two CFA models

	Model 1	Model 2
***P*** **-value (chi-square)**	0.000	0.002
**CFI**	0.851	0.950
**TLI**	0.780	0.922
**RMSEA**	0.120^a^	0.071^b^

^a^ with 90% confidence interval 0.094–0.147

^b^ with 90% confidence interval 0.041–0.101

**Fig. 1:**
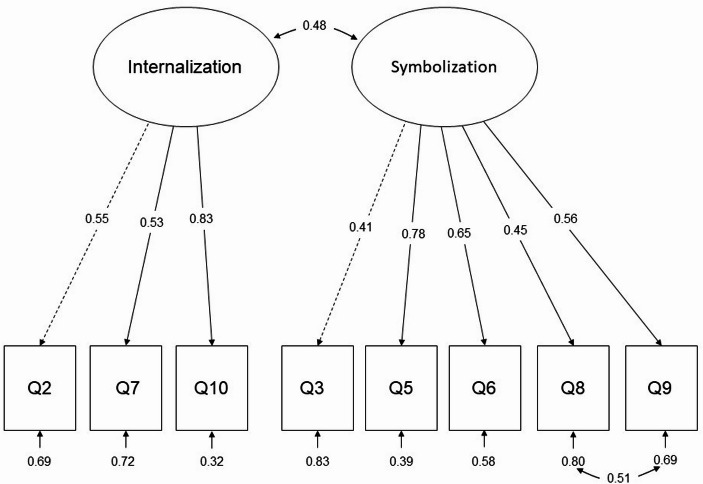
Final CFA model of EPMI (Model 2)

Figure 1 shows the CFA results of Model 2. The figure shows two latent variables (Internalization and Symbolization) and eight measured variables (Q2, Q7, Q10, Q3, Q5, Q6, Q8, and Q9).

## Discussion

In this discussion section, we will first discuss the validity of the EPMI based on the results of the factor analyses we conducted. After that, we will discuss how EPMI can complement the existing engineering ethics assessment instruments and how EPMI can contribute to engineering ethics education.

### EPMI Validity Discussion

While EPMI needs further validation, our results provide positive initial validity evidence of the measure. The two-factor structure suggested by the original moral identity of Aquino and Reed (2002) could also be replicated with the modified items, evidenced by the exploratory and confirmatory factor analysis results. This indicates the modified items continue to reflect the two underlying dimensions of moral identity – internalization and symbolization – although we had to drop two items (Q1 and Q4) before conducting the confirmatory factor analysis.

The items Q1 (“It would make me feel good to be an engineer who has these characteristics”) and Q4 (“I would be ashamed to be an engineer who has these characteristics”) showed both strong negative skewness (Q1’s skewness was -4.26, and Q4’s skewness was -4.29) and very high kurtosis (Q1’s kurtosis was 23.54, and Q4’s kurtosis was 18.62). This indicates potential ceiling effect (Garin, 2024), which means most participants strongly endorsed those items by selecting mostly strongly agree/agree (in Q4’s case, strongly disagree/disagree), leaving little variability in their responses.

A potential explanation could be that the participants in this study are engineering professionals, and the values discussed in EPMI are emphasized in engineering professional codes of ethics. While values in Aquino and Reed (2002)’s original measure are all generally considered as characteristics that are good to have, they are not systematically emphasized through formal education, as engineering education, which is a professional education, emphasizes engineering ethics codes in their curriculum. Therefore, we could say that EPMI, which was developed by modifying Aquino and Reed (2002)’s moral identity measure to better fit the engineering context, can perform better with 8 items.

The remaining 8 items loaded adequately on their intended factors. However, as Fig. 1 shows, two items (Q3, “I often dress in ways that identify me as having these characteristics”, and Q8, “The fact that I have these characteristics is communicated to others by my membership in certain organizations”) showed somewhat low factor loadings (lower than 0.50). While we decided to retain these items in EPMI to preserve consistency with the original moral identity measure, future studies may consider revising them to make them better contribute to the intended factor.

### EPMI for Engineering Ethics Assessment

Current ethics assessment instruments in engineering education, such as the Engineering and Science Issues Test (ESIT) and the Engineering Ethical Reasoning Instrument (EERI), focus primarily on students’ ethical reasoning abilities. As Kim and Bairaktarova (2023) note, most existing ethics assessment instruments focus on cognitive skills such as moral reasoning or understanding of codes, but they fail to capture whether students see ethical responsibility as integral to their professional self-concept. However, they do not assess students’ motivations or whether they view ethics as part of who they are as engineers. This is where the EPMI measure adds value; it can assess whether ethical responsibility is internalized as part of a student’s professional identity.

The inclusion of identity as an assessment domain is important because research suggests that individuals are more likely to behave ethically when ethical values are central to their self-concept (Aquino & Reed, 2002). While tools like the ESIT or EERI can indicate whether students understand ethical issues and moral reasoning, they do not reveal whether students are inclined to act ethically under pressure or in ambiguous circumstances. EPMI can complement them by linking ethical understanding to professional identity, offering insight into whether students view ethical behavior as part of “what engineers do” and “who engineers are.” This identity-driven perspective is essential for cultivating engineers who will maintain ethical standards even in the absence of external incentives or oversight.

Kim and Bairaktarova (2023) also emphasize that many instruments, such as the Engineering Professional Responsibilities Assessment (ERPA) and Civics-Minded Graduate Scale (CMG), evaluate students’ beliefs about ethics or their understanding of professional responsibilities. However, again, these tools do not directly measure whether students feel personally committed to ethical action. EPMI can complement those measures by assessing the degree to which students regard ethical engineering practice as central to their identity. This adds a critical layer to ethics assessment because students who internalize ethical values are more likely to demonstrate consistent ethical behavior across different professional contexts.

Another advantage of EPMI is its potential for longitudinal assessment. Administering EPMI at multiple points can help educators observe how students come to see themselves as ethical professionals over time. Longitudinal assessment is also crucial for identifying when and how students experience shifts in their ethical reasoning or begin to internalize professional values, as these are developmental processes that unfold over time (Colby & Sullivan, 2008).

### EPMI for Engineering Ethics Education

Integrating the EPMI measure into engineering ethics education creates a pathway for teaching ethics as a core component of professional identity. The EPMI framework will be able to support students’ development into professionals who identify as responsible engineers. By focusing on identity, EPMI complements existing instruments by addressing the identity dimensions of ethical development that are not captured by reasoning-focused tools.

Fostering moral identity formation requires moving beyond cognitive skill-focused teaching strategies. As Herkert (2000) notes, ethical engagement is most effective when students see themselves as moral agents operating within a complex professional landscape. Reflective practices such as journaling, personal narratives, and values-driven discussion (Hitt et al., 2020; Vanello, 2025; Woodson & Zhu, 2018; McOwen et al., 2025) can help students articulate how their ethical beliefs intersect with their emerging professional identity. EPMI can be used by educators to reflect on how such activities contribute to the internalization of ethical values and provide insight into whether students are simply learning about ethics or beginning to view ethical responsibility as central to their engineering identity.

Additionally, EPMI enables educators to differentiate instruction based on students’ varying degrees of moral identity integration. Particularly if based on longitudinal assessment, educators can evaluate the long-term effects of ethics interventions, such as mentoring programs, service-learning, or research ethics training. Collecting several rounds of EPMI measurements can provide evidence of moral identity development as students progress through the curriculum, particularly if ethics-driven interventions and experiences take place in the classroom (Kim & Bairaktarova, 2023).

As Hess et al. (2024) make apparent, a one-size-fits-all approach to ethics education is unlikely to meet the needs of a diverse student body that varies in terms of culture, engagement strategy, and perspective. EPMI can help identify where students need support and assist instructors in designing targeted learning experiences. For example, students with lower moral identity scores might benefit from structured opportunities to explore the social consequences of engineering work through various self-reflective activities (Whitbeck, 2011; Hersh, 2016) and case studies focusing on macroethical challenges, such as the societal impacts of information and communication technologies and AI (Floridi, 2014, 2023). Meanwhile, students with higher EPMI scores may be equipped to take on more autonomous forms of ethical engagement, leading classroom discussions on ethics (McDonald et al., 2022) or engaging in community-based engineering projects (Nieusma & Riley, 2010), which require more independent judgment and social awareness. By aligning instructional strategies with students’ ethical development, educators can better support the formation of engineers who are both technically capable and ethically grounded.

## Conclusion

The Engineering Professional Moral Identity (EPMI) measure is a tool designed to assess the degree to which ethical responsibility is integrated into an individual’s sense of self as an engineer. Unlike traditional ethics assessments that focus on cognitive reasoning or rule comprehension, EPMI targets the motivational and identity-based dimensions of ethical engagement. In the context of engineering ethics education, an EPMI measure can serve as a valuable tool to evaluate how students internalize ethical values and perceive their role in upholding public welfare, safety, and professional integrity. This approach aligns with broader calls in literature to move ethics instruction from abstract theorizing toward identity-centered development (Martin et al., 2021).

Beyond assessment, EPMI provides a foundation for designing more personalized and developmentally appropriate ethics instruction. Students vary widely in how they understand and embody ethical responsibility, and EPMI can help educators identify where learners are in their development. Additionally, integrating EPMI into engineering ethics education reframes ethics not as a checklist of correct decisions but as an evolving part of who students are becoming as professionals. It allows for longitudinal assessment of identity formation and offers insight into whether ethics interventions produce sustained changes in how students perceive their responsibilities as engineers. Over time, repeated EPMI assessments can help track the effectiveness of curricular strategies such as mentoring, service learning, and reflective writing.

While the EPMI instrument requires further validation, the positive preliminary evidence supports this identity-centered approach and affirms its promise as a novel tool for engineering ethics assessment. By centering identity and emphasizing the internalization of moral responsibility, EPMI addresses a crucial gap left by traditional instruments focused on reasoning and principles application. Continued refinement and empirical testing will enhance the EMPI’s utility. Ultimately, EPMI provides a powerful framework that can be used to cultivate engineers who not only understand what ethical behavior entails, but who also view ethical responsibility as integral to their professional self-concept to the point of action. In doing so, it offers a meaningful step toward fostering a generation of engineers who are not only technically proficient but morally grounded and committed to advancing the public good.

## Appendix

### Engineering Professional Moral Identity Measure Items and Instruction

#### Instruction

We are interested in how engineers and other technical professionals think about who they are. Listed below are some characteristics that might describe an engineer:

honest; high integrity; cooperative; loyal; conscientious; objective; technically competent; fair-minded; committed to quality; open to criticism; committed to public health, safety, and welfare.

The engineer with these characteristics could be you or it could be someone else. For a moment, visualize in your mind the kind of engineer who has these characteristics. Imagine how that engineer would think, feel, and act. When you have a clear image of what this engineer would be like, answer the following questions.

#### Items

1. It would make me feel good to be an engineer who has these characteristics.
2. Being an engineer who has these characteristics is an important part of who I am.
3. I often dress in ways that identify me as having these characteristics.
4. I would be ashamed to be an engineer who has these characteristics. **(R)**
5. The types of things I do in my spare time (e.g., hobbies, volunteering) clearly identify me as having these characteristics.
6. The kinds of media I consume identify me as having these characteristics.
7. Having these characteristics is not really important to me. **(R)**
8. The fact that I have these characteristics is communicated to others by my membership in certain organizations.
9. I am actively involved in activities that communicate to others that I have these characteristics.
10. I strongly desire to have these characteristics.

#### Factors

Internalization − 1, 2, 4, 7, 10.

Symbolization − 3, 5, 6, 8, 9.

##### Scale

5-point Likert scale from strongly disagree (1) to strongly agree (5).

1 - strongly disagree.

2 - disagree a little.

3 - neither agree nor disagree.

4 - agree a little.

5 - strongly agree.

## Data Availability

Data cannot be shared openly to protect the privacy of human subjects.

## Code Availability

R code is available from the corresponding author upon reasonable request.
